# Editorial: Harnessing invasive plant species into valuable products

**DOI:** 10.3389/fpls.2026.1913523

**Published:** 2026-06-30

**Authors:** Bhaskar Sarma, Garima Singh, Guannan Wang, Kalidas Upadhyaya, Bindu Yadav

**Affiliations:** 1 Department of Botany, Dhemaji College, Dhemaji, Assam, India; 2 Department of Botany, Pachhunga University College, Mizoram University, Aizawl, India; 3 Department of Biology, Stanford University, Stanford, CA, United States; 4 Howard Hughes Medical Institute, Stanford University, Stanford, CA, United States; 5 Department of Forestry, Mizoram University, Aizawl, India; 6 School of Environmental Sciences, Jawaharlal Nehru University (JNU), New Delhi, India

**Keywords:** biomass valorisation, circular bioeconomy, environmental remediation, resource recovery, sustainable development

## 1 Introduction

Invasive plant species (IPS) represent one of the most important drivers of biodiversity loss, ecosystem degradation and agricultural productivity decline worldwide. They grow fast, are widely adapted ecologically and are competitive, enabling them to invade new habitats, outcompete native vegetation and modify ecosystem functioning, often with considerable ecological and economic costs. Invasive plants have long been considered ecological liabilities that need constant vigilance, containment, and eradication (Pyšek et al., 2020). Recent developments, however, have started to challenge this traditional view. Many invasive plants produce large amounts of biomass, and exhibit varied phytochemicals, metabolites and structural compounds that have potential applications in environmental remediation, agriculture, industrial biotechnology, pharmaceuticals and renewable energy production (Young et al., 2011; Rai and Kim, 2020). This new perspective is consistent with the principles of the circular bioeconomy, which seeks to transform biological resources into value-added products with reduced waste and environmental footprints.

**Figure 1:**
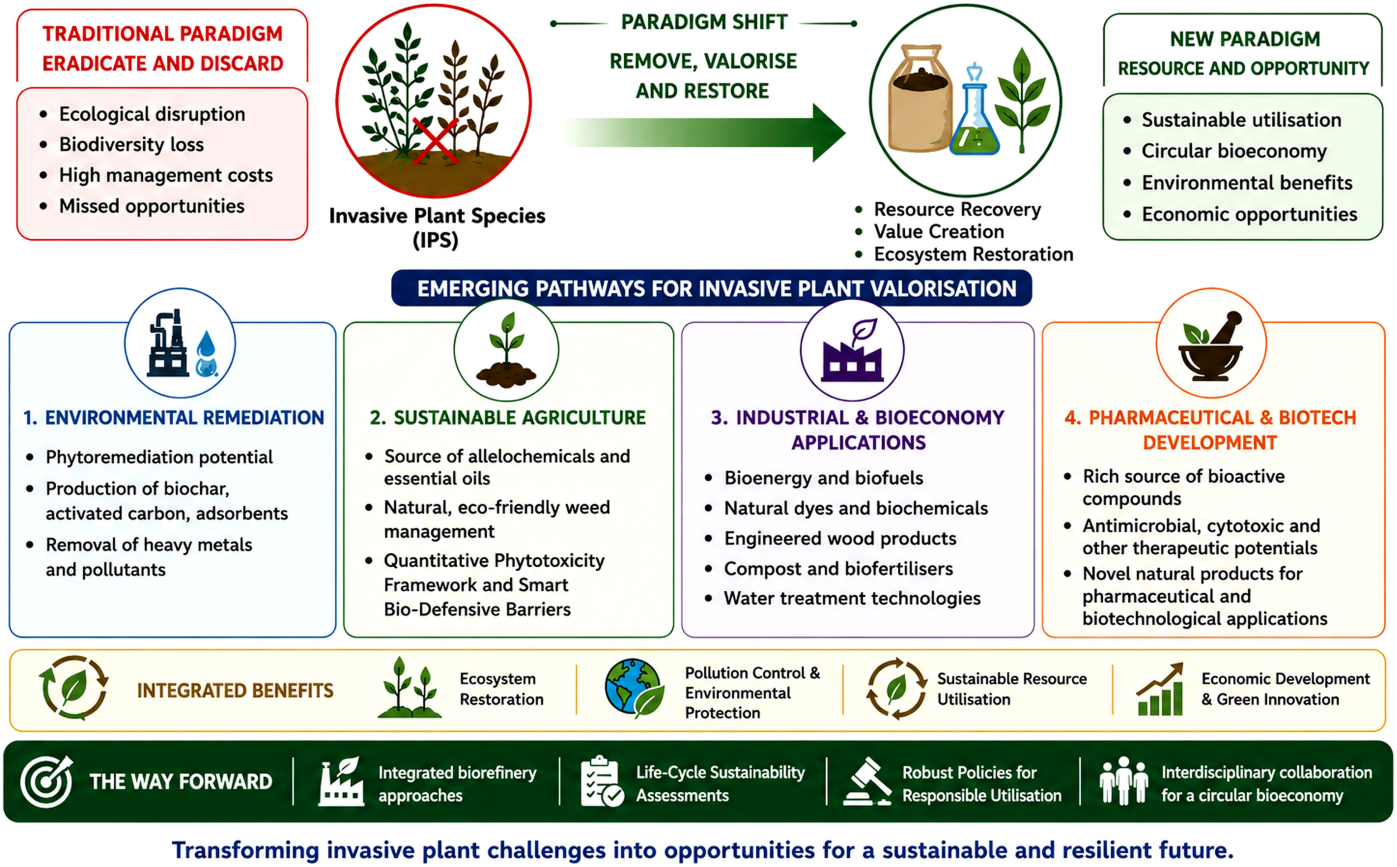
Conceptual framework illustrating the transition from conventional invasive plant management toward invasive plant valorisation within a circular bioeconomy framework.

The transition from a “eradicate and discard” to a “remove, valorise, and restore” paradigm is a major paradigm shift in invasion science. Research is increasingly focused on how to convert invasive biomass into valuable resources that generate environmental, social and economic benefits, rather than simply viewing it as a management problem. Against this larger scientific context, the Research Topic *Harnessing Invasive Plant Species into Valuable Products* was created to encourage innovative strategies for the conversion of invasive plant biomass into useful products and promote ecosystem restoration and sustainable development (**Figure 1**).

## 2 Invasive plant species: from ecological burden to valuable biological resources

Historically, invasive species management has been about prevention, containment and eradication (Tobin, 2018). These methods remain necessary, but they tend to ignore the large resource potential of invasive biomass. Across many regions, invasive plants generate large quantities of biomass that are usually removed and discarded, adding to management costs and missed opportunities for resource recovery. Growing interest in valorisation of invasive plants is part of a broader trend towards sustainable resource management. Similar to agricultural residues and forestry wastes, invasive biomass could be a renewable feedstock for bio-based materials, bioenergy, speciality chemicals, and natural products. This perspective does not discount the ecological hazards of invasive species; rather it integrates management goals with resource recovery approaches. The studies assembled in this Research Topic collectively demonstrate, how invasive biomass can contribute to environmental sustainability, agricultural resilience, industrial innovation and pharmaceutical development.

### 2.1 Emerging pathways for invasive plant valorisation

One of the most promising applications of invasive biomass is for environmental remediation. Biochar, activated carbon and engineered adsorbents derived from plant materials are becoming recognised as sustainable tools for pollution control and resource recovery. In this context, Mohanty et al. have indicated the potential of invasive bamboo and bamboo derived products for the chromium remediation. Their work demonstrates how invasive biomass can be employed as a phytoremediation agent and as a source of remediation materials to remove contaminants from soil and water systems. Such approaches are examples of how invasive species management can contribute to both environmental restoration and value creation.

Innovative solutions may come from invasive plants in another sector is agriculture. It is believed that the ecological success of many invasive species is due to the production of allelochemicals that inhibit competing vegetation. These compounds are valuable sources of natural bioactive molecules that can be utilised for sustainable management of weeds. Sabrine and Benmeddour systematic review and meta-analysis provide quantitative evidence of the phytotoxic potential of invasive plant-derived essential oils. Their results show the potential of these natural products as eco-friendly weed management tools and present new concepts such as Quantitative Phytotoxicity Framework and Smart Bio-Defensive Barriers. Such approaches exemplify the potential to exploit ecological traits associated with invasion for sustainable agricultural purposes.

The circular bioeconomy aims to extract the maximum value from biological resources by efficient use and resource recovery. In this context, invasive plant biomass is an abundant but unexploited feedstock. Patil et al. investigate the potential for valorisation of globally important invasive species such as *Lantana camara*, *Prosopis juliflora*, *Leucaena leucocephala*, *Acacia mearnsii*, and *Senna spectabilis*. Their assessment shows the versatility of invasive biomass for such uses as bioenergy, natural dyes, engineered wood products, composts, pharmaceuticals and water treatment technology. Crucially, their work underscores a change in philosophy in invasive species management, away from expensive control and towards resource-generating systems that enable both ecological restoration and economic development.

The pharmaceutical and biotech sectors are among the highest value opportunities for invasive plant valorisation. Many invasive species have unique metabolic adaptations leading to the production of special bioactive compounds with potential industrial and medical applications. This potential has been shown by Borska et al. in their study of *Heracleum sosnowskyi* where bioactive lipid compounds with antimicrobial and cytotoxic properties were identified. Their results support Sathe increasing awareness that invasive species may be promising sources of novel natural products for pharmaceutical and biotechnological applications.

## 3 Conclusion

The contributions compiled in this Research Topic emphasise a major shift in current invasion science, from considering invasive plant species as mere targets to be eradicated, to seeing them as potential resources for sustainable bioeconomy schemes. Collectively, the studies demonstrate that invasive plant biomass can be utilised for a range of purposes from environmental remediation and sustainable agriculture to industrial biotechnology and pharmaceutical development, thus transforming management challenges into opportunities for resource recovery and value creation. This new paradigm does not reduce the ecological risks of biological invasions, but instead promotes the integration of responsible management practices and innovative utilisation strategies. As global efforts shift towards circular economy principles and sustainable resource use, the valorisation of invasive plants offers a promising route to foster ecological restoration, environmental sustainability and economic development simultaneously. The challenge ahead lies in ensuring that utilization approaches remain scientifically robust, economically viable, and ecologically responsible.

## References

[B1] Pyšek P. Hulme P. E. Simberloff D. Bacher S. Blackburn T. M. Carlton J. T. . (2020). Scientists' warning on invasive alien species. Biol. Rev. Cambridge Philos. Soc. 95, 1511–1534. doi: 10.1111/brv.12627 32588508 PMC7687187

[B2] Rai P. K. Kim K.-H. (2020). Invasive alien plants and environmental remediation: a new paradigm for sustainable restoration ecology. Restor. Ecol. 28, 3–7. doi: 10.1111/rec.13058 40046247

[B3] Tobin P. C. (2018). Managing invasive species. F1000Research 7, F1000 Faculty Rev-1686. doi: 10.12688/f1000research.15414.1 30416712 PMC6206619

[B4] Young S. L. Gopalakrishnan G. Keshwani D. R. (2011). Invasive plant species as potential bioenergy producers and carbon contributors. J. Soil Water Conserv. 66, 45A–50A. doi: 10.2489/jswc.66.2.45A

